# Implementing a clinical pharmacy intervention for older adult inpatients with chronic non-cancer pain: a feasibility study

**DOI:** 10.1007/s11096-025-02033-8

**Published:** 2025-10-18

**Authors:** Jasmin Abderhalden, Céline Lang, Carla Meyer-Massetti, Danja Müller, Patricia Cadisch, Dominic Bertschi, Aljoscha Noël Goetschi

**Affiliations:** 1 Clinical Pharmacology and Toxicology, Department of General Internal Medicine, Inselspital, Bern University Hospital, Anna-Von-Krauchthal-Weg 7, 3010 Bern, Switzerland; 2 Institute of Primary Health Care (BIHAM), University of Bern, 3012 Bern, Switzerland; 3 Institute for Hospital Pharmacy, Bern University Hospital, 3010 Bern, Switzerland; 4 Department of Geriatrics, Bern University Hospital, 3010 Bern, Switzerland; 5 Graduate School for Health Sciences, University of Bern, 3012 Bern, Switzerland

**Keywords:** Chronic pain, Clinical pharmacy, Older adults, Medication safety, Feasibility study, Interprofessional collaboration

## Abstract

**Introduction:**

Chronic non-cancer pain (CNCP) affects 22–88% of older adults and is associated with a lower quality of life and polypharmacy. It thus puts these already very vulnerable patients at a greater risk of medication-related harm.

**Aim:**

This feasibility study aimed to implement a multimodal clinical pharmacy intervention to improve CNCP-related care for older adult inpatients on hospital geriatrics wards.

**Method:**

We conducted a single-arm feasibility study from January to May 2025, including patients aged 65 or older, hospitalised on the geriatrics ward of a tertiary hospital in Switzerland and previously diagnosed with CNCP. Feasibility was defined as the ability to perform the intervention as planned and approximated by recruitment and dropout rates. The intervention included semi-structured interviews about patients’ pain histories, collected patient-reported outcome measures (PROMs) and recorded therapy goals. Pharmacists then conducted medication reviews using a previously developed and validated trigger tool. The trigger tool was used as a standardised approach for identifying medication-related issues, comprising a set of previously validated quality indicators. Findings were discussed during interprofessional ward rounds. Final treatment decisions were made jointly with patients. We followed up with patients by telephone one month after hospital discharge.

**Results:**

Of 253 screened patients, we included 48 patients: 28 (58%) were interviewed, and 18 (38%) had a follow-up telephone call. Pharmacists suggested 56 therapy changes, with 29 identified by the trigger tool and 27 identified by regular medication review. Therapy change acceptance rates by the care team were 78% and 41%, respectively. Pain frequency and the highest and lowest pain levels over the last seven days all decreased after hospital discharge, although these changes cannot be causally attributed to the intervention. Other pain-related PROMs showed no change or just a slight improvement or deterioration.

**Conclusion:**

The present feasibility study showed that implementing a clinical pharmacy intervention for older adult inpatients was indeed feasible. However, the recruitment rates were relatively low, and dropout rates were relatively high. Using a standardised approach involving a trigger tool showed promising results for detecting medication-related problems. These are important first indicators that including pharmacists more closely in standard care could be beneficial to CNCP patients.

**Supplementary Information:**

The online version contains supplementary material available at 10.1007/s11096-025-02033-8.

## Impact statements

- Combined with a variety of other rehabilitation measures for older adults, a specialised clinical pharmacy intervention may contribute to improving patient-relevant outcomes.
- This feasibility study showed that improved interprofessional collaboration on CNCP management is possible between nurses, physicians and clinical pharmacists. However, the acceptance rates of pharmacist interventions suggest that there is still room for improvement in this collaboration.
- Clinical pharmacists can help increase medication safety by combining a standardised trigger tool with personalised, patient-centred clinical reasoning.
- This feasibility study laid the groundwork for future larger studies, including (cluster)-randomised clinical trials to evaluate the effects of clinical pharmacy interventions among older adult populations with CNCP.

## Introduction

Chronic non-cancer pain (CNCP) is defined as pain, not caused by cancer, that persists or recurs for more than three months [1]. Although CNCP is common in the general population, it affects older adults more, with 28–88% of them suffering from CNCP [2]. Newer prevalence studies report that 30–38% of older adults suffer from CNCP [3, 4]. The higher frequency of musculoskeletal and joint conditions in older adults particularly influences this high prevalence of CNCP [5]. It is noteworthy that up to 88% of people with CNCP suffer from multiple other chronic diseases [6, 7]. Depression and insomnia are especially common comorbidities, with both exerting a negative influence on CNCP and both being influenced by CNCP themselves [8–10]. To control their CNCP and treat its comorbidities, older adults often use multiple medications, known as polypharmacy [11, 12]. Even if polypharmacy can be appropriate, it is well known to be closely linked to occurrences of adverse drug events [13, 14], such as medication errors, drug–drug interactions or adverse drug reactions [15]. To increase medication safety, pharmacists have been striving to improve CNCP care.

Three systematic reviews have demonstrated the potential benefits of pharmacist-led interventions to manage CNCP. A 2011 systematic review and meta-analysis reported that pharmacist-led patient education, such as group-based or individualised approaches, reduced pain intensity [16]. A 2014 review focusing on medication reviews (MRs) by pharmacists found they led to lower pain levels [17]. More recently, a systematic review and meta-analysis confirmed these positive effects for a broader range of pharmacist interventions, beyond education and MRs and including interprofessional interventions [18]. However, the interventions described in those reviews focused on the general population, not older adults. While clinical pharmacists have demonstrated the potential benefits of them working directly on geriatrics wards [19–21], a recent scoping review (including all study designs to give a more detailed depiction of clinical pharmacy practices related to CNCP) failed to identify any clinical pharmacy interventions specifically targeting older adults with CNCP [22].

In Switzerland, there are relatively few clinical pharmacists per hospitalised patients [23], and specialised clinical pharmacy interventions remain rare. To identify medication-related problems, pharmacists conduct MRs which require time and personnel resources [24]. Another approach to detecting MRPs is the use of trigger tools. These tools use a set of quality indicators (QIs) as triggers to flag patients. Reviewers then assess these patients for potential MRPs. Scanning electronic patient records for triggers can be automated, meaning that reviewers only have to manually assess flagged patients [25]. As electronic algorithms provide rapid insight, they generate statistics on quality performance and provide the opportunity to intervene in a targeted manner [25]. In addition, trigger tools that use QIs require fewer resources than full MRs while remaining efficient [24]. Therefore, trigger tools based on validated QIs might help clinical pharmacists to become more active in CNCP care.

### Aim

Using a previously validated set of QIs as a trigger tool [26, 27], this proof-of-concept study aimed to implement a clinical pharmacy intervention for older adult inpatients with CNCP hospitalised on an acute geriatrics ward in a university hospital in Switzerland.

## Method

### Study design

We conducted a five-month, single-group feasibility study to test a novel clinical pharmacy MR intervention from January to May 2025. We followed the Consolidated Standards of Reporting Trials (CONSORT) extension to randomised pilot and feasibility trials in reporting [28] (Supplementary file 1).

### Intervention

**Fig. 1:**
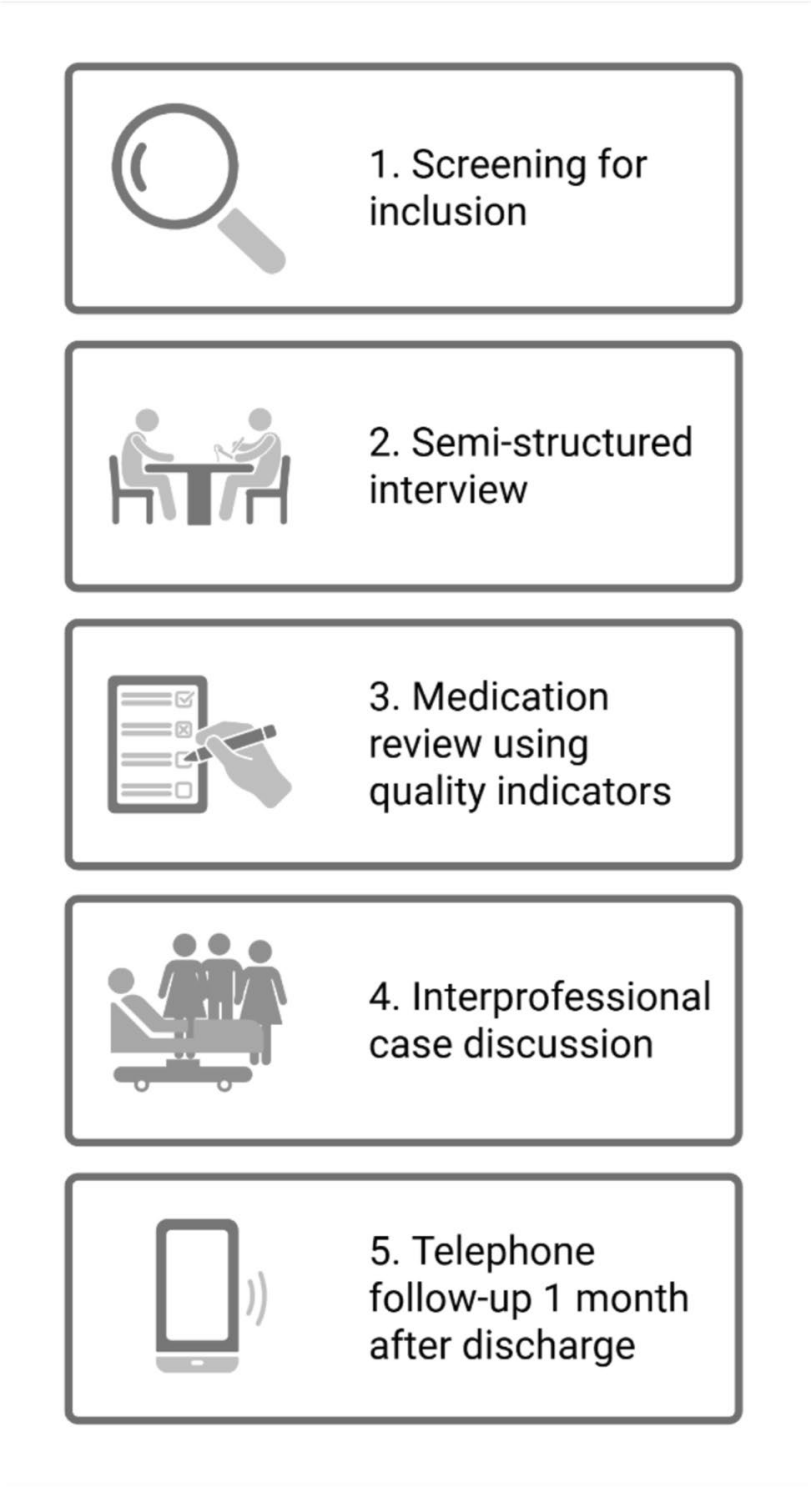
Graphical depiction of the clinical pharmacy intervention tested. Created with BioRender.com

Our clinical pharmacy intervention comprised five parts: 1. patient screening, 2. patient interview, 3. medication analysis, 4. interprofessional discussion of the case, and 5. telephone follow-up (see Fig. 1). First, every Monday, we screened the charts of newly admitted patients for diagnoses of CNCP. Second, we conducted semi-structured interviews with them, including noting their pain history, clarifying their treatment goals, and discussing adverse effects and the effectiveness of their current treatments, which were usually established prior to hospital admission. Interviews also sought patient-reported outcome measures (PROMs) based on the Initiative on Methods, Measurement, and Pain Assessment in Clinical Trials (IMMPACT) core outcomes set [29] and the World Health Organization Disability Assessment Schedule 2.0 [30]. Specifically, the interviews included questions on mobility, the basic activities of daily living (ADLs), the instrumental activities of daily living (IADLs), sleep quality and emotional impairment due to pain. These outcomes were recorded using a 5-point (0–4) Likert scale (see Supplementary File 2 for the translated interview guide). Third, we carried out comprehensive MRs, including standard evaluations of patients’ prescribed drugs [31]. In addition, the clinical pharmacists also used a set of previously developed QIs [26, 27]. These QIs were designed to help clinical pharmacists efficiently identify MRPs in older adults with CNCP (see Supplementary File 3 for a list of the QIs used). Fourth, the clinical pharmacists discussed suggested therapy changes with the patients’ physicians and nurses (care team) during an interprofessional ward round. Final treatment decisions were made together with the patients. Fifth, one month after their hospital discharge, we contacted patients who had completed the semi-structured interview for a second semi-structured interview via telephone, focused on the same factors touched upon in the first interview. All data were collected in a REDCap® database, which is stored on a secure server.

### Participants

We included inpatients hospitalised in the University Hospital of Bern’s geriatrics clinic, in Switzerland, if they were ≥ 65 years old, had at least one diagnosis of CNCP and had a planned discharge date not before Friday of the week their screening was conducted. We excluded patients with any cancer-related pain. The assigned resident physician assessed whether patients were cognitively capable of participating in an interview. If they were not, we included them in the overall intervention but did not interview them.

### Outcomes

The primary outcome was an evaluation of whether the clinical pharmacy intervention developed was feasible, which we defined as the ability to successfully perform our clinical pharmacy intervention as planned [32]. We approximated feasibility by describing the number of patients recruited and the dropout rates per intervention stage [33]. The secondary outcome was recording a set of PROMs, including pain intensity and frequency, mobility, restrictions to performing the ADLs and IADLs, sleep quality, and emotional impairments due to pain. The tertiary outcome was a description of the interventions made by our clinical pharmacists.

### Statistical methods

We used descriptive statistics to describe the study population and their PROMs. This included calculating medians and interquartile ranges (IQRs) or percentages, as appropriate. We created violin plots to show the distribution of PROMs before and one month after the intervention. Data analysis was conducted with R, using the packages ‘tidyverse’ and ‘Tableone’ [34–36].

### Ethics approval

This study was exempt from full ethics approval by the Canton of Bern’s Ethics Committee because it met the criteria for a quality improvement study (Req-2024-01252). All participating patients signed a general waiver consenting to the further use of their data.

## Results

### Feasibility assessment

**Fig. 2:**
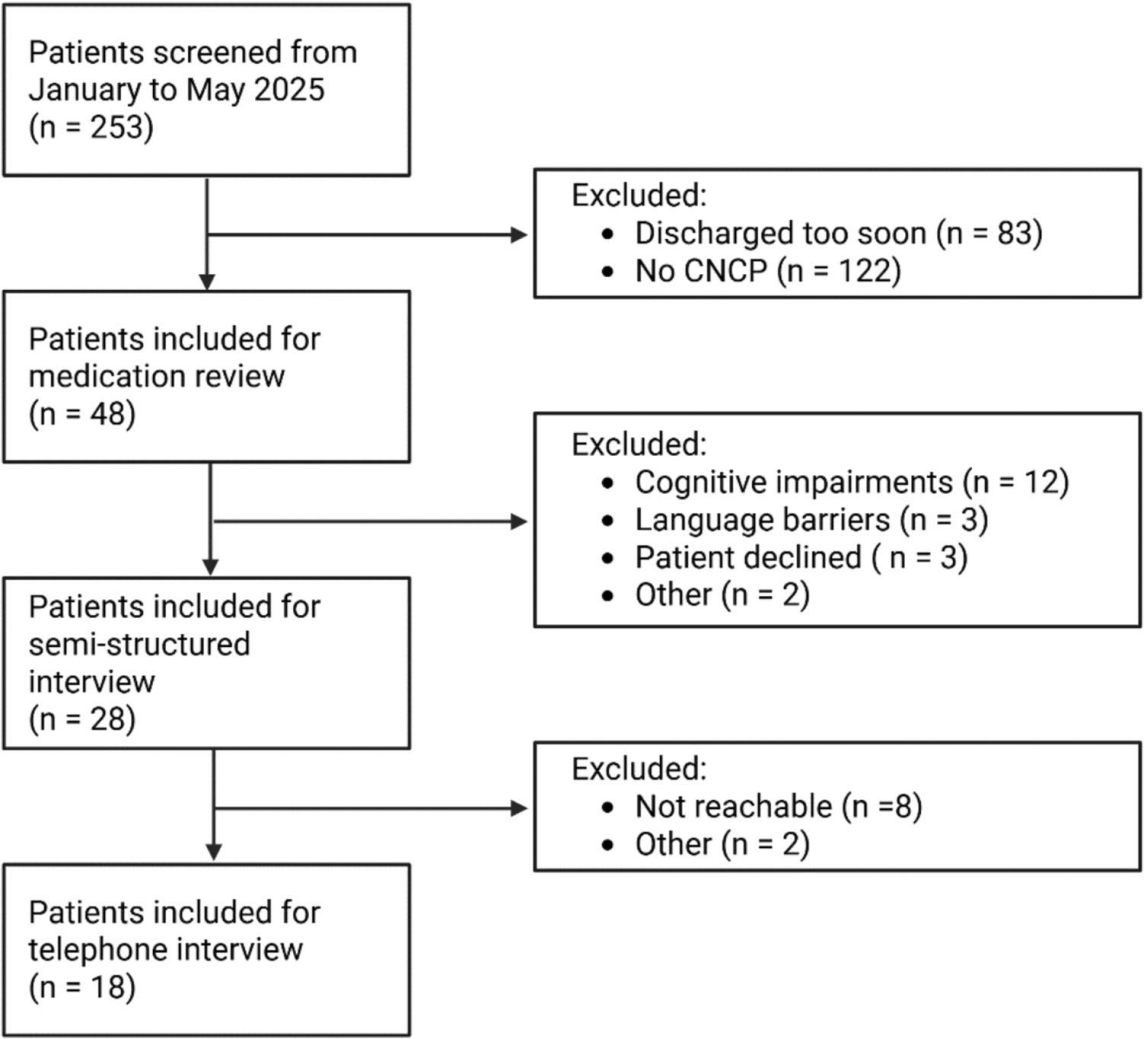
Patients included and excluded at each stage of the study. CNCP = chronic non-cancer pain. Created with BioRender.com

During the five-month pilot phase, we successfully implemented the clinical pharmacy services as planned. We screened 253 admissions, and 48 older adult inpatients with CNCP underwent an MR. Of the 48 patients, 28 (58%) were interviewed using a semi-structured approach. The main reason for interviews being impossible was the high prevalence of cognitive impairment (e.g. dementia and delirium). Of the 28 interviewed patients, 18 (64%) underwent telephone follow-up. The main reason for dropping out at this stage was that we could not contact the patients. Figure 2 is a flow chart showing participant inclusion at each stage.

### Patient characteristics

The median patient was 83 years old (IQR: 80–90), and 28 were women (58%). Patients took a median of 11 drugs regularly (IQR: 8–13) and a further median of 8 ‘as needed’ drugs (IQR: 7–9), as defined in the electronic patient charts. Pain medications consisted predominantly of paracetamol (n = 45, 94%) and metamizole (n = 42, 88%). Half of the patients took opioids (n = 24, 50%). Chronic low back pain was the most common CNCP diagnosis (n = 31, 65%), followed by arthritis (n = 22, 46%) and neuropathic pain (n = 11, 23%). More information is shown in Table 1.

**Table 1:** Overview of patient characteristics

Characteristics	Number of patients n = 48
Age, median [IQR]	83 [80, 90]
Sex = female, n (%)	28 (59)
Normal place of residence = at home, n (%)	46 (96)
Drugs, median [IQR]	11 [8, 13]
Drugs PRN, median [IQR]	8 [7, 9]
Paracetamol, n (%)	45 (94)
NSAIDs, n (%)	2 (4)
Topical NSAIDs, n (%)	8 (17)
Metamizole, n (%)	42 (88)
Opioids, n (%)	24 (50)
SNRIs, n (%)	2 (4)
TCAs, n (%)	1 (2)
Diagnoses, median [IQR]	20 [17, 23]
Back pain, n (%)	31 (65)
Arthritis, n (%)	22 (46)
Rheumatoid arthritis, n (%)	6 (13)
Headache, n (%)	2 (4)
Neuropathic pain, n (%)	11 (23)

IQR = interquartile range; NSAIDs = non-steroidal anti-inflammatory drugs; PRN = pro re nata; SNRIs = serotonin noradrenaline reuptake inhibitors; SMD = standardised mean difference; TCAs = tricyclic antidepressants

### Pharmacists’ interventions

In total, the pharmacists suggested 56 improvements to the patients’ CNCP therapies, corresponding to a median of 1 intervention (IQR: 0–2) per patient. Of these 56 suggestions, 26 (47%) resulted from the standard MR, and a further 29 (53%) were derived from the new QIs and the trigger tool. Of all 56 suggested actions, 33 (59%) were accepted by the care team and 20 (36%) were not. Decisions on six interventions (11%) were deferred (usually until consultation with the primary care physician). Of the interventions resulting from the standard MR, 11 were accepted (41%), 3 were deferred (11%), and 13 were not accepted (48%). Of the 29 interventions derived from our new QIs, 22 were accepted by the care team (78%), 3 were deferred (10%), and 4 were not accepted (14%). More information can be seen in Tables 2 and 3.

**Table 2:** Overview of the pharmacists’ suggested interventions resulting from the standard MR

Suggested intervention	Status	Number (%) n = 27
Stop potentially inadequate medications	Accepted Not accepted	3 (11%) 1 (4%)
Increase dose	Accepted	3 (11%)
Stop duplicated drugs	Accepted	2 (7%)
Stop drugs without indications	Accepted	1 (4%)
Stop treatment because of inappropriate duration	Accepted Decision deferred	1 (4%) 1 (4%)
Decrease dose	Accepted	1 (4%)
Stop contraindicated drug	Decision deferred	1 (2%)
Mitigate drug–drug interactions	Decision deferred	1 (4%)
Add missing treatment	Not accepted	8 (30%)
Add missing documentation in the patient’s chart	Not accepted	2 (7%)
Resolve adverse drug event	Not accepted	2 (7%)

For each QI, we detail how many interventions were accepted, deferred for consultation or rejected by the care team. We only show statuses that had associated cases

**Table 3:** Overview of the pharmacists’ suggested interventions derived from the new quality indicators (QIs)

QI	Suggested intervention	Status	Number (%) n = 29
G8	Add topical therapy for localised CNCP	Accepted Decision deferred Not accepted	9 (31%) 1 (3%) 2 (7%)
P1	Decrease the daily paracetamol dose to under 3 g	Accepted	4 (14%)
G3	Resolve adverse drug events	Accepted Not accepted	2 (7%) 1 (3%)
O6	Add bowel regimen to opioid therapy	Accepted	2 (7%)
M1	Stop metamizole in those with a higher risk for agranulocytosis	Accepted Decision deferred	1 (3%) 2 (7%)
N2	Stop NSAIDs in cases of renal impairment	Accepted	1 (3%)
N7	Stop NSAIDs in cases of heart failure	Accepted	1 (3%)
N8	Stop NSAIDs with glucocorticoid therapy	Accepted	1 (3%)
O14	Stop sedative comedications with opioid therapy	Accepted	1 (3%)
C1	Start co-analgesics in cases of neuropathic pain	Not accepted	1 (3%)

For each QI, we detail how many interventions were accepted, deferred for consultation or rejected by the care team. We only show statuses that had associated cases

### Patient-reported outcome measures

**Fig. 3:**
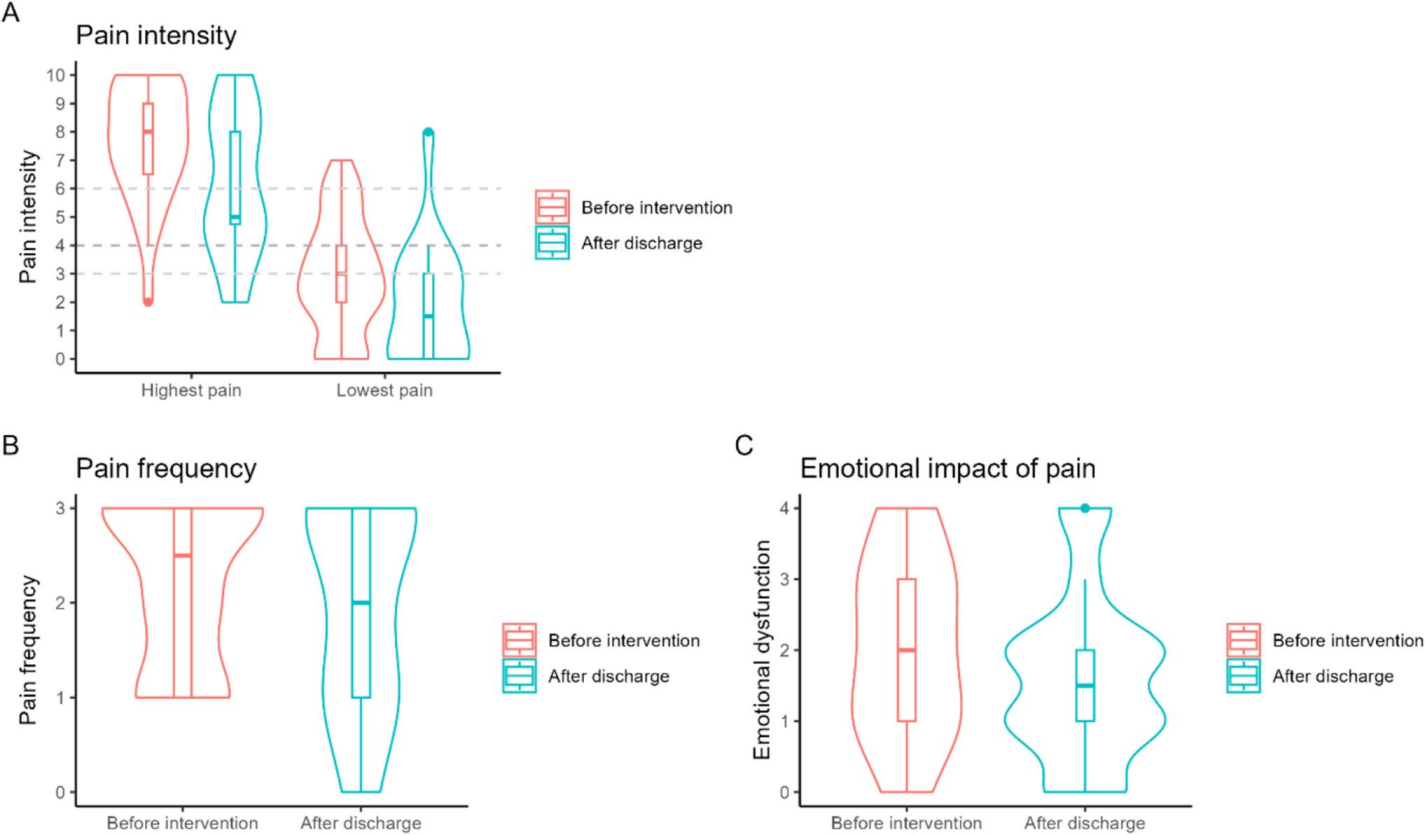
Violin and box plots of pain-related patient-reported outcome measures before pharmacists’ interventions and one month after hospital discharge. Graph **A** shows the highest and lowest pain intensities in the seven days before the interviews. The grey dotted lines indicate the median and interquartile range of patient-reported acceptable pain levels. Graph **B** shows pain frequency, with 0 = never, 1 = some days, 2 = most days and 3 = every day. Graph **C** shows the emotional impact that pain has, with 0 = none, 1 = low impact, 2 = moderate impact, 3 = strong impact and 4 = very strong impact

**Fig. 4:**
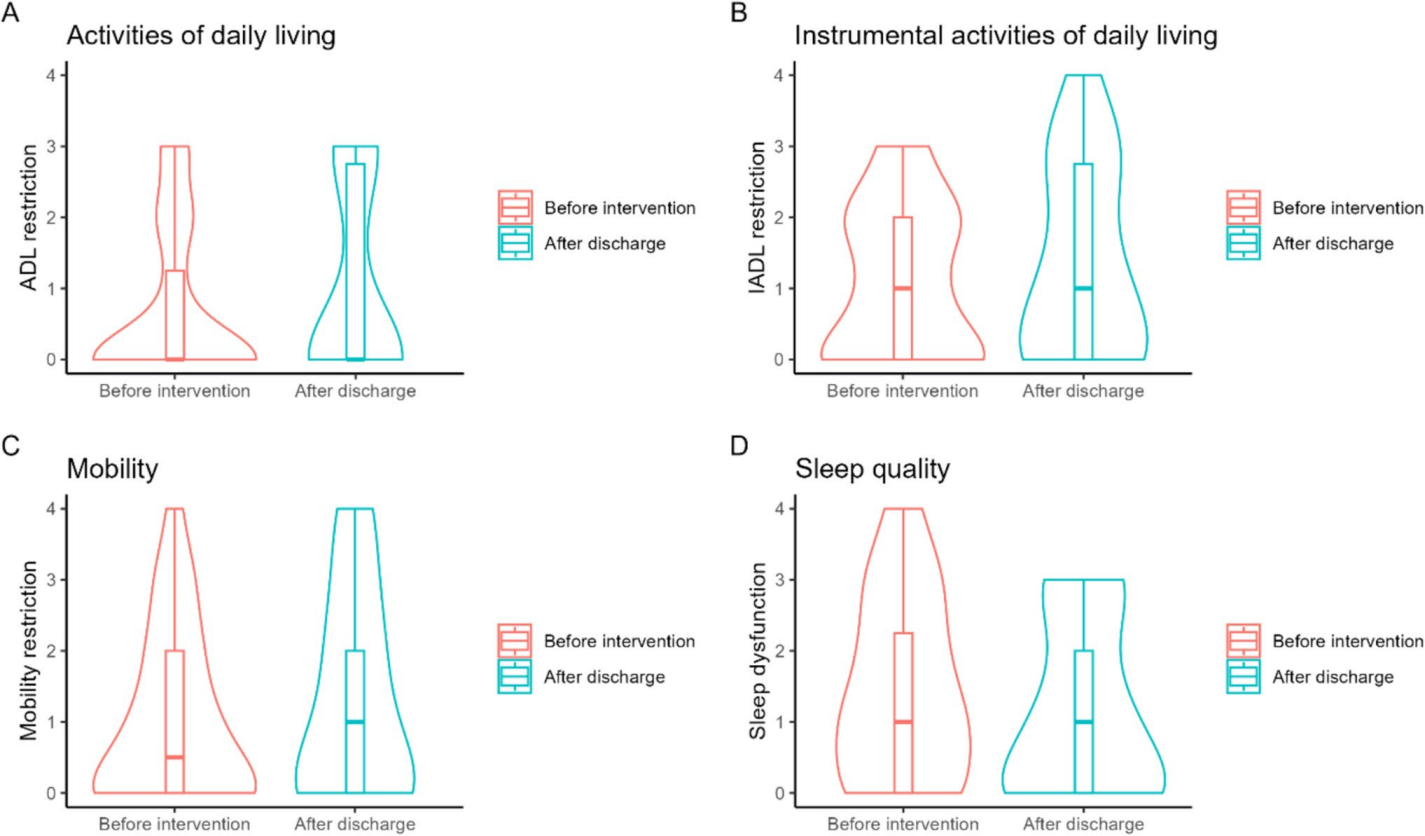
Comparison of patient-reported outcome measures before pharmacists’ interventions and one month after hospital discharge. The scales correspond to 0 = no restriction, 1 = low restriction, 2 = moderate restriction, 3 = strong restriction and 4 = very strong restriction. ADL: activities of daily living; IADL: instrumental activities of daily living

Compared to patients before the intervention, patients after the intervention reported lower pain-related PROMs after discharge. The median frequency of pain decreased from 2.5 (IQR: 1–3) to 2 (IQR: 1–3), with 3 corresponding to pain every day and 2 to pain on most days. The highest level of pain in the last few days, on a scale from 0 to 10, decreased from 8 (IQR: 6.5–9) to 5 (IQR: 4.75–8), and the lowest level of pain decreased from 3 (IQR: 2–4) to 1.5 (IQR: 0–3). The median level of the emotional impact of pain decreased from 2 (IQR: 1–3) to 1.5 (IQR: 1–2), with 2 corresponding to a moderate impact and 1 to a low impact. We observed no significant changes in patients’ median levels of sleep quality or restrictions in their ability to complete the basic ADLs and IADLS. The median mobility level deteriorated, however, from 0.5 (IQR: 0–2) to 1 (IQR: 0–2), with 0 corresponding to no restrictions and 1 to low restrictions to mobility. The changes in these PROMs levels are shown in Figs. 3 and 4. Before the intervention, 9 of 28 patients interviewed (32%) reported that their therapy goals were currently met. After discharge, 12 out of 18 (67%) believed they had met their therapy goals. Overall, the completeness of PROMs data collection was in the median 98% (IQR: 96–100%) during the patient interview and 85% (IQR: 89–100%) during the telephone follow-up.

## Discussion

### Statement of key findings

This five-month, single-group study demonstrated the feasibility of a novel clinical pharmacy intervention for older adult inpatients with CNCP. Of the 253 patients originally screened, 19% were recruited for an enhanced MR, 11% also underwent a patient interview, and 7% responded to telephone follow-up one month after hospital discharge. Therefore, 58% of patients were interviewed and 64% were followed up by telephone. One month after discharge, patients reported lower pain-related outcomes, such as their highest and lowest levels of pain in the seven days before the interview, intensity of pain and the emotional impact of pain. Other PROMs remained the same, including patients’ sleep quality and restrictions in their ability to complete the ADLs and IADLS. Their post-discharge mobility deteriorated slightly, however. Compared to a third of patients before the intervention, two thirds of patients after it had met their therapy goals one month after their discharge. Pharmacists intervened a median of once per patient. The care team accepted 59% of pharmacists’ suggested interventions following a standard MR; however, they accepted 78% of the intervention suggestions derived from our QIs. Frequently suggested interventions were related to improving topical therapies for localised CNCP (e.g. topical diclofenac), optimising paracetamol dosages or mitigating adverse drug events.

### Strengths and weaknesses

The present study’s main strength was its use of a multimodal, interprofessional clinical pharmacy intervention. To the best of our knowledge, this was the first study to test such an intervention for older adults—a patient population disproportionally affected by CNCP [1] and often excluded from trials [37]. Another strength was the outcomes chosen for evaluation, which map onto the IMMPACT core outcomes set [29] and the World Health Organization Disability Assessment Schedule 2.0 [30]. Finally, the trigger tool selected for the study was based on a validated set of QIs able to efficiently identify relevant MRPs, and this yielded high acceptance rates by the care team.

The study had some limitations, nevertheless. First, such a single-arm feasibility study could have serious implications on the interpretation of our findings. For example, improvements in PROMs cannot be attributed solely to the clinical pharmacy intervention but also to the comprehensive rehabilitation programme available on the geriatrics ward, including physiotherapy, occupational therapy, social interventions and dietary counselling. Second, roughly one third of patients were lost to follow-up after discharge. This may have introduced some attrition bias, as the patients not involved in the study may have been sicker. Third, the study might have been subject to implementation bias, meaning that the environment was more controlled and ideal than the real world. When implementing the intervention in routine care, more barriers might come to light.

### Interpretation

This study demonstrated the feasibility of a clinical pharmacy intervention for older adult inpatients with CNCP. However, the overall recruitment rate was relatively low compared to the expected prevalence of CNCP of 22–88% [1], which could be due to several factors, including a lower CNCP prevalence than expected in our population at the time of the study or different CNCP diagnosis criteria. Overall, only 19% of the patients admitted to our geriatrics ward had their medication reviewed, and 11% underwent an in-house patient interview. The main reason for this was that we required at least a one-week length of stay to conduct our intervention, and many patients were discharged before this was possible. Weekly ward rounds, including clinical pharmacists, and other ward practices somewhat constrained us.

However, the study revealed that, in combination with other geriatrics rehabilitation measures, a clinical pharmacy intervention could contribute to improving PROMs. This aligns with other pharmacist interventions for patients with CNCP, including educational interventions [16], an MR alone [17] and diverse interventions involving multimodal management [18]. However, those studies did not focus on older adult patients. Systematic reviews of clinical pharmacy interventions have found a reduction in pain intensity (e.g. standardised mean difference [SMD]: − 0.22; 95% confidence interval [95%CI]: − 0.35 to − 0.09) corresponding to a small effect [17, 18, 38]. One systematic review found no improvement in physical function [18], while another reported an SMD of − 0.38 (95%CI: − 0.58 to − 0.18) [17], also corresponding to a small effect [38]. For other outcomes, including quality of life, a meta-analysis was impossible. Acknowledging the fact that we cannot measure the isolated effect of the clinical pharmacy intervention tested, the reductions in pain levels and other PROMs are similar to those reported in other studies in the literature.

The care team accepted 59% of the CNCP management interventions suggested by pharmacists, a rate comparable to other Swiss studies that have tested general clinical pharmacy interventions. In a 2019 study focusing on patients in a general internal medicine unit, 57.6% of interventions were accepted [39], and in a 2015 study, also conducted in general internal medicine, an initial acceptance rate of 84% of recommendations was reported, but only 58% of interventions were actually implemented [40]. Importantly, the acceptance rate of the pharmacists’ suggestions derived from our QIs was higher, reaching 78%. This reinforces our hypothesis that using a trigger tool—developed using guideline methods involving a systematic literature search and a consensus-building method, such as the RAND/UCLA Delphi study [41]—can help clinical pharmacists formulate interventions acceptable to both patients and the care team. Of course, as previous research has shown, a trigger tool can never replace a full MR by a pharmacist, which includes verifying dosages, drug–drug interactions, contraindications and therapy guidelines specific to the patient [42, 43]. The fact that around half of the proposed therapy changes arose during the MR underlines this. However, the limited acceptance rate of MR-derived interventions (41%) could impact patient safety, highlighting the need for improved interprofessional communication.

### Further research

Future research should continue to evaluate this promising clinical pharmacy intervention. It should involve using a more robust design, ideally a (cluster)-randomised clinical trial. To increase the patient participation rate, the intervention timeline should be made more flexible, and a longer follow-up period should be evaluated, including analysing whether patients adhere to their medication plans, the effects of clinical pharmacists’ interventions persist, outcomes are maintained and pain-related PROMs continue to improve. This trial could also include subgroup analyses to evaluate the effect of the intervention on different types of CNCP. To minimise loss-to-follow-up after patient discharge, novel techniques, such as automated telephone follow-ups, should be explored [44], given their potential benefits [45, 46]. In addition, a qualitative study could explore the reasons behind the care team’s and the patients’ decisions to accept, defer or decline pharmacist interventions. Future studies should also make a cost–benefit evaluation of this specialised clinical pharmacy intervention, including an evaluation of the time spent by pharmacists on interviews, MRs and telephone follow-up. More generally, it is important to continue researching the role of clinical pharmacists in geriatrics and CNCP.

## Conclusion

The present single-arm feasibility study demonstrated that a clinical pharmacy intervention for older adult inpatients with chronic non-cancer pain (CNCP) hospitalised in a tertiary hospital geriatrics ward in Switzerland, was indeed feasible. This study also suggested that, in combination with other geriatrics rehabilitation measures, involving clinical pharmacists in CNCP care may improve patient-relevant outcomes.

## Supplementary Information

Below is the link to the electronic supplementary material.

Supplementary file1 (DOC 228 KB)

Supplementary file2 (PDF 94 KB)

Supplementary file3 (PDF 124 KB)

## Data Availability

The datasets used and/or analysed during the current study are available from the corresponding author upon reasonable request.

## References

[1] Treede R-D, Rief W, Barke A, et al. Chronic pain as a symptom or a disease: the IASP Classification of Chronic Pain for the International Classification of Diseases (ICD-11). Pain. 2019;160(1):19–27. 30586067 10.1097/j.pain.0000000000001384

[2] Helme RD, Gibson SJ. The epidemiology of pain in elderly people. Clin Geriatr Med. 2001;17(3):417–31. 11459713 10.1016/s0749-0690(05)70078-1

[3] Rikard SM, Strahan AE, Schmit KM, et al. Chronic pain among adults—United States, 2019–2021. MMWR Morb Mortal Wkly Rep. 2023;72(15):379–85. 37053114 10.15585/mmwr.mm7215a1 PMC10121254

[4] LaRowe LR, Miaskowski C, Miller A, et al. Prevalence and sociodemographic correlates of chronic pain among a nationally representative sample of older adults in the United States. J Pain. 2024;25(10). 10.1016/j.jpain.2024.104614 PMC11402580 38936750

[5] Urwin M, Symmons D, Allison T, et al. Estimating the burden of musculoskeletal disorders in the community: the comparative prevalence of symptoms at different anatomical sites, and the relation to social deprivation. Ann Rheum Dis. 1998;57(11):649–55. 9924205 10.1136/ard.57.11.649 PMC1752494

[6] Mills SEE, Nicolson KP, Smith BH. Chronic pain: a review of its epidemiology and associated factors in population-based studies. Br J Anaesth. 2019;123(2):e273–83. 31079836 10.1016/j.bja.2019.03.023 PMC6676152

[7] Scherer M, Hansen H, Gensichen J, et al. Association between multimorbidity patterns and chronic pain in elderly primary care patients: a cross-sectional observational study. BMC Fam Pract. 2016;17(1):68. 27267905 10.1186/s12875-016-0468-1 PMC4895952

[8] Jank R, Gallee A, Boeckle M et al. Chronic pain and sleep disorders in primary care. Pain Res Treat. 2017;2017. 10.1155/2017/9081802 PMC5749281 29410915

[9] Banks SM, Kerns RD. Explaining high rates of depression in chronic pain: a diathesis-stress framework. Psychol Bull. 1996;119(1):95.

[10] Currie SR, Wang J. More data on major depression as an antecedent risk factor for first onset of chronic back pain. Psychol Med. 2005;35(9):1275–82. 16168150 10.1017/S0033291705004952

[11] Goetschi AN, Verloo H, Wernli B, et al. Prescribing pattern insights from a longitudinal study of older adult inpatients with polypharmacy and chronic non-cancer pain. Eur J Pain 28(10):1645–55. 10.1002/ejp.2298 38838067

[12] Jebara T, Youngson E, Drummond N, et al. A qualitative exploration of chronic pain management of older adults in remote and rural settings. Int J Clin Pharm. 2023;45(6):1405–14. 37392351 10.1007/s11096-023-01607-8 PMC10682030

[13] Just KS, Dormann H, Böhme M, et al. Personalising drug safety—results from the multi-centre prospective observational study on Adverse Drug Reactions in Emergency Departments (ADRED). Eur J Clin Pharmacol. 2020;76(3):439–48. 31832731 10.1007/s00228-019-02797-9

[14] Dubrall D, Just KS, Schmid M, et al. Adverse drug reactions in older adults: a retrospective comparative analysis of spontaneous reports to the German Federal Institute for Drugs and Medical Devices. BMC Pharmacol. 2020;21(1):25. 10.1186/s40360-020-0392-9 PMC7092423 32293547

[15] Nebeker JR, Barach P, Samore MH. Clarifying adverse drug events: a clinician’s guide to terminology, documentation, and reporting. Ann Intern Med. 2004;140(10):795–801. 15148066 10.7326/0003-4819-140-10-200405180-00009

[16] Bennett MI, Bagnall A-M, Raine G, et al. Educational interventions by pharmacists to patients with chronic pain: systematic review and meta-analysis. Clin J Pain. 2011;27(7):623–30. 21610491 10.1097/AJP.0b013e31821b6be4

[17] Hadi MA, Alldred DP, Briggs M, et al. Effectiveness of pharmacist-led medication review in chronic pain management: systematic review and meta-analysis. Clin J Pain. 2014;30(11):1006–14. 24480911 10.1097/AJP.0000000000000063

[18] Thapa P, Lee SWH, Kc B, et al. Pharmacist-led intervention on chronic pain management: a systematic review and meta-analysis. Br J Clin Pharmacol. 2021;87(8):3028–42. 33486825 10.1111/bcp.14745

[19] Simal I, Capiau A, De Spiegeleer A, et al. Evaluating an integrated clinical pharmacist model in a geriatric day hospital: a prospective single-centre observational study. Int J Clin Pharm. 2025. 10.1007/s11096-025-01934-y. 10.1007/s11096-025-01934-y 40465185

[20] Montaleytang M, Correard F, Spiteri C, et al. Medication reconciliation in the geriatric unit: impact on the maintenance of post-hospitalization prescriptions. Int J Clin Pharm. 2021;43(5):1183–90. 33464484 10.1007/s11096-021-01229-y

[21] Kiesel EK, Drey M, Pudritz YM. Influence of a ward-based pharmacist on the medication quality of geriatric inpatients: a before–after study. Int J Clin Pharm. 2022;44(2):480–8. 35076810 10.1007/s11096-021-01369-1 PMC9007813

[22] Goetschi AN, Meyer-Massetti C. Characterising pharmacists’ interventions in chronic non-cancer pain care: a scoping review. Int J Clin Pharm. 2024;46(5):1010–23. 38861043 10.1007/s11096-024-01741-x PMC11399199

[23] Studer H, Boeni F, Messerli M, et al. Clinical pharmacy activities in swiss hospitals: How have they evolved from 2013 to 2017? Pharmacy. 2020;8(1):19. 32046296 10.3390/pharmacy8010019 PMC7151693

[24] Meyer-Massetti C, Cheng CM, Schwappach DLB, et al. Systematic review of medication safety assessment methods. Am J Health Syst Pharm. 2011;68(3):227–40. 21258028 10.2146/ajhp100019

[25] Murphy DR, Meyer AN, Sittig DF, et al. Application of electronic trigger tools to identify targets for improving diagnostic safety. BMJ Qual Saf. 2019;28(2):151–9. 30291180 10.1136/bmjqs-2018-008086 PMC6365920

[26] Goetschi AN, Verloo H, Schönenberger N, et al. Quality indicators for the pharmacological management of chronic non-cancer pain in older adult patients: an integrative review. J Eval Clin Pract. 31(5):e70253. 10.1111/jep.70253 PMC12365588 40831201

[27] Goetschi AN, Schönenberger N, Wernli U, et al. Developing quality indicators for the pharmacological management of chronic non-cancer pain in older adult inpatients: a RAND/UCLA Delphi study. J Pain Res. 2025;18:5037–56. 10.2147/JPR.S533027 PMC12487695 41041662

[28] Eldridge SM, Chan CL, Campbell MJ, et al. CONSORT 2010 statement: extension to randomised pilot and feasibility trials. BMJ. 2016;355:i5239. 27777223 10.1136/bmj.i5239 PMC5076380

[29] Turk DC, Dworkin RH, Allen RR, et al. Core outcome domains for chronic pain clinical trials: IMMPACT recommendations. Pain. 2003;106(3):337–45. 14659516 10.1016/j.pain.2003.08.001

[30] World Health Organization. WHODAS II-Disability assessment schedule training manual: A guide to administration. Geneva: World Health Organization. 2000. 978 92 4 154759 8.

[31] Griese-Mammen N, Hersberger KE, Messerli M, et al. PCNE definition of medication review: reaching agreement. Int J Clin Pharm. 2018;40(5):1199–208. 30073611 10.1007/s11096-018-0696-7

[32] Karsh BT. Beyond usability: designing effective technology implementation systems to promote patient safety. Qual Saf Health Care. 2004;13(5):388. 15465944 10.1136/qshc.2004.010322 PMC1743880

[33] Proctor E, Silmere H, Raghavan R, et al. Outcomes for implementation research: conceptual distinctions, measurement challenges, and research agenda. Adm Policy Ment Health. 2011;38(2):65–76. 20957426 10.1007/s10488-010-0319-7 PMC3068522

[34] R Development Core Team. R: A language and environment for statistical computing. Vienna, Austria: R Foundation for Statistical Computing;2022.

[35] Wickham H, Averick M, Bryan J, et al. Welcome to the tidyverse. J Open Source Softw. 2019;4(43):1686.

[36] Yoshida K, Bartel A. Tableone: Create'Table 1'to describe baseline characteristics with or without propensity score weights. R package version 0.13.2 ed2014.

[37] Zulman DM, Sussman JB, Chen X, et al. Examining the evidence: a systematic review of the inclusion and analysis of older adults in randomized controlled trials. J Gen Intern Med. 2011;26(7):783–90. 21286840 10.1007/s11606-010-1629-x PMC3138606

[38] Chittaranjan A. Mean difference, standardized mean difference (SMD), and their use in meta-analysis: as simple as it gets. J Clin Psychiatry. 2020;81(5):11349. 10.4088/JCP.20f13681 32965803

[39] Reinau D, Furrer C, Stämpfli D, et al. Evaluation of drug-related problems and subsequent clinical pharmacists’ interventions at a Swiss university hospital. J Clin Pharm Ther. 2019;44(6):924–31. 31408206 10.1111/jcpt.13017

[40] Guignard B, Bonnabry P, Perrier A, et al. Drug-related problems identification in general internal medicine: The impact and role of the clinical pharmacist and pharmacologist. Eur J Intern Med. 2015;26(6):399–406. 26066400 10.1016/j.ejim.2015.05.012

[41] Campbell SM, Braspenning J, Hutchinson A et al. Research methods used in developing and applying quality indicators in primary care. Qual Saf Health Care 2002;11(4):358–64. 10.1136/qhc.11.4.358 PMC1758017 12468698

[42] Karpov A, Parcero C, Mok CPY, et al. Performance of trigger tools in identifying adverse drug events in emergency department patients: a validation study. Br J Clin Pharmacol. 2016;82(4):1048–57. 27279597 10.1111/bcp.13032 PMC5137830

[43] Noorda NMF, Sallevelt B, Langendijk WL, et al. Performance of a trigger tool for detecting adverse drug reactions in patients with polypharmacy acutely admitted to the geriatric ward. Eur Geriatr Med. 2022;13(4):837–47. 35635713 10.1007/s41999-022-00649-x PMC9378479

[44] Harrison JD, Sudore RL, Auerbach AD, et al. Automated telephone follow-up programs after hospital discharge: Do older adults engage with these programs? J Am Geriatr Soc. 2022;70(10):2980–7. 35767470 10.1111/jgs.17939 PMC9588657

[45] Odeh M, Scullin C, Fleming G, et al. Ensuring continuity of patient care across the healthcare interface: telephone follow-up post-hospitalization. Br J Clin Pharmacol. 2019;85(3):616–25. 30675742 10.1111/bcp.13839 PMC6379220

[46] Svahn S, Gallego G, Gustafsson M, et al. Geriatric patients’ views on a pharmacist-led follow-up programme after discharge from hospital. Explor Res Clin Soc Pharm. 2025;18:100597. 40275943 10.1016/j.rcsop.2025.100597 PMC12018084

