# Supplementary material for: Implementing a clinical pharmacy intervention for older adult inpatients with chronic non-cancer pain: a feasibility study
Source: Int J Clin Pharm. 2025 Oct 18;48(2):597–606. doi: 10.1007/s11096-025-02033-8 (PMC12992365; doi:10.1007/s11096-025-02033-8)
Supplement: Supplementary file 2 — Supplementary file2 (PDF 94 KB) [file 11096_2025_2033_MOESM2_ESM.pdf]

Patient interview
Hospital

Record ID ______________________

Feasibility of the interview

The patient is able according to care/AA to conduct a short interview.
- ◯ Yes
- ◯ No

Interview conducted?
- ◯ Yes
- ◯ No

Why was the interview not conducted?
- ◯ Patient could not be located
- ◯ Patient did not want to participate in the
- ◯ interview Other reason

Other reason? ______________________

Graded chronic pain scale

https://pmc.ncbi.nlm.nih.gov/articles/PMC7097879/

In the last 3 months, how often have you Pain?
- ◯ Never
- ◯ Some days
- ◯ Most days Every day
- ◯

How often have you experienced pain in the last three months restricted your life or work?
- ◯ Never
- ◯ Some days
- ◯ Most days Every day
- ◯

In the last 7 days, how severe has your pain been on average on a scale of 0-10?

Or average NRS of the last days from EPIC.
- ◯ 0
- ◯ 1
- ◯ 2
- ◯ 3
- ◯ 4
- ◯ 5
- ◯ 6
- ◯ 7
- ◯ 8
- ◯ 9
- ◯ 10

Which number best describes how much the Have you experienced pain in the last 7 days in your impaired your enjoyment of life?	◯ 0<br>◯ 1<br>◯ 2<br>◯ 3<br>◯ 4<br>◯ 5<br>◯ 6<br>◯ 7<br>◯ 8<br>◯ 9<br>◯ 10
Which number best describes how much the pain in the last 7 days has affected your general activity in the last 7 days?	◯ 0<br>◯ 1<br>◯ 2<br>◯ 3<br>◯ 4<br>◯ 5<br>◯ 6<br>◯ 7<br>◯ 8<br>◯ 9<br>◯ 10
PEG score (questions 3, 4 and 5: total)	______________________
GCPS score<br><br>Grade 0: No chronic pain<br><br>Grade 1: Mild chronic pain<br><br>Grade 2: bothersome chronic pain<br><br>Grade 3: severe pain	______________________

Brief medication history (medication at home!)

To be completed before the consultation, to be checked during the consultation.

The first 3 questions are about pain medication that is taken regularly.

Which pain medication does the patient take regularly?<br><br>Use the ATC code!	______________________
What is the daily dose (in mg)?	______________________
Which pain medication is the patient taking as a fixed dose?<br><br>Use ATC code!	______________________
What is the daily dose (in mg)?	______________________

Which pain medication is the patient taking?<br><br>Use ATC code!	______________________
What is the daily dose (in mg)?	______________________
The next 3 questions are about back-up pain medication.	
Which back-up pain medication is the patient taking?<br><br>Use ATC code!	______________________
What is the daily dose (in mg)?	______________________
Which back-up pain medication is the patient taking?<br><br>Use ATC code!	______________________
What is the daily dose (in mg)?	______________________
Which back-up pain medication is the patient taking?<br><br>Use ATC code!	______________________
What is the daily dose (in mg)?	______________________
How often do you need your reserve medication?	◯ daily<br>◯ several times a week<br>◯ once a week several<br>◯ times a month<br>◯ less than once a month never<br>◯
Now some general questions about medication.	
Do you sometimes forget to take your medication?	◯ Yes<br>◯ No
Are you sometimes careless about your medication?<br>taking your medication?	◯ Yes<br>◯ No
When you feel better, do you sometimes<br>stop taking your medication?	◯ Yes<br>◯ No
If you feel worse after taking your<br>medication, do you sometimes stop taking it?<br>do you sometimes stop taking them?	◯ Yes<br>◯ No

Adherence score

____

Willingness to adhere

1= high willingness to adhere

0= low willingness to adhere

____

Have you experienced any side effects from your pain medication?

Ask openly and, if the answer is no, discuss the typical ADRs of the medication taken.

- ◯ Yes
- ◯ No

What ADRs?

____

Now a question about other treatments/multimodal therapy

What non-drug treatments are you currently using for your for your pain are you currently doing?

- ☐ physical therapies (physio, massages etc.)
- ☐ Psychological therapies
- ☐ Acupuncture, TENS
- ☐ Other treatments

Some questions on patient satisfaction

What is your therapy goal regarding your pain? QoL,

measurable things, not just intensity...

____

Does your current treatment fulfill this goal?

- ◯ Yes
- ◯ No

Why not and what needs to change?

____

Are there already things from the interview that could be adjusted?

____

Would you benefit from more support in the treatment of your pain?

- ◯ Yes
- ◯ No

Would it help you if you had more information about your pain medication?

What are side effects? How can I deal with them?

- ◯ Yes
- ◯ No

Give information to the patient: Discuss possible solutions with the treating medical team --> next Thursday we would talk about this again.

We would like to give you the following information after you leave hospital again to see how things are going with your pain is going well. Is that OK?

- ◯ Yes
- ◯ No
