# Supplementary material for: Implementing a clinical pharmacy intervention for older adult inpatients with chronic non-cancer pain: a feasibility study
Source: Int J Clin Pharm. 2025 Oct 18;48(2):597–606. doi: 10.1007/s11096-025-02033-8 (PMC12992365; doi:10.1007/s11096-025-02033-8)
Supplement: Supplementary file 3 — Supplementary file3 (PDF 124 KB) [file 11096_2025_2033_MOESM3_ESM.pdf]

No.	Quality Indicator
G1	IF an older adult has CNCP, THEN define care goals with the patient that focus on quality of life and functionality.
G3	IF an older adult has CNCP, THEN monitor for adverse drug events.
G5	IF an older adult has CNCP, THEN pharmacological treatment should be provided.
G6	IF an older adult is being treated for CNCP, THEN choose oral drugs.
G8	IF an older adult has localised CNCP, THEN use topical drugs.
G9	IF an older adult has CNCP, THEN use sustained release forms around the clock.
G10	IF an older adult is diagnosed with CNCP, THEN use base medication combined with as-needed medication.
O5	IF an older adult with CNCP is treated using opioids, THEN their effects should be monitored.
O6	IF an older adult with CNCP is treated using opioids, THEN they should be offered a bowel regimen or medical records should document the potential for constipation or explain why bowel treatment is not needed.
O7	IF an older adult with CNCP requires opioids, THEN pethidine/meperidine should not be used.
O9	IF an older adult with CNCP is treated using opioids, THEN use long-acting formulations.
O10	IF an older adult with CNCP is treated using opioids, THEN use short-acting opioids for breakthrough pain.
O11	IF an older adult with CNCP is treated using opioids THEN they should have tried all other options without success.
O12	IF an older adult with CNCP and renal impairment is treated using opioids, THEN consider using buprenorphine, hydromorphone or oxycodone.
O13	IF an older adult with CNCP requires opioids, THEN avoid codeine.
O14	IF an older adult with CNCP is treated using opioids, THEN other sedative drugs should be avoided.
O16	IF an older adult with CNCP and hepatic impairment is treated using opioids, THEN consider hydromorphone.
O17	IF an older adult with CNCP requires opioids, THEN do not use tramadol.
N1	IF an older adult with CNCP<br>- has a history of peptic ulcer disease or gastrointestinal bleeding and/or<br>- currently uses antithrombotics, anticoagulants, corticosteroids or SSRIs<br>AND they are treated using a cyclooxygenase-nonselective NSAID[3], THEN they should be provided concomitant treatment with a proton pump inhibitor.
N2	IF an older adult with CNCP has renal impairment, THEN do not use NSAIDs.
N3	IF an older adult with CNCP has peptic ulcers or gastrointestinal bleeding, THEN do not use NSAIDs.
N6	IF an older adult with CNCP is treated using NSAIDs, THEN do not use multiple NSAIDs.
N7	IF an older adult with CNCP has heart failure or other cardiovascular diseases, THEN do not use NSAIDs.
N8	IF an older adult with CNCP is treated using NSAIDs, THEN do not combine with corticosteroids.
N9	IF an older adult with CNCP requires NSAIDs, THEN do not use indomethacin.
N10	IF an older adult with CNCP has an H. pylori infection, THEN do not use NSAIDs.
N11	IF an older adult with CNCP requires NSAIDs, THEN do not use ketorolac.
P1	IF an older adult with CNCP is treated using paracetamol, THEN do not exceed a dose of 3 g per day.
P2	IF an older adult with CNCP and liver cirrhosis is treated using paracetamol, THEN avoid daily doses above 2 g.
P3	IF an older adult with CNCP and chronic alcohol consumption is treated using paracetamol, THEN avoid daily doses above 2 g.
M1	IF an older adult has CNCP and low blood granulocyte counts or other agranulocytosis-inducing drugs, THEN avoid metamizole.
M2	IF an older adult with CNCP is started on metamizole, THEN educate and monitor the patient for symptoms of agranulocytosis.

C1	IF an older adult has neuropathic CNCP, THEN consider co-analgesics.
C2	IF an older adult with CNCP has closed-angle glaucoma, benign prostate hyperplasia, urinary retention, constipation, cardiovascular diseases or severe hepatic disease, THEN avoid TCAs[4].
C3	IF an older adult has CNCP, THEN avoid TCAs.
C4	IF an older adult with CNCP is treated using TCAs, THEN avoid high doses (e.g. no more than 10–25 mg of amitriptyline).
C5	IF an older adult has CNCP, THEN do not use carbamazepine.
C6	IF an elder with CNCP requires an SNRI, THEN prefer Duloxetine to Venlafaxine.

CNCP: chronic non-cancer pain; NSAIDs: non-steroidal anti-inflammatory drugs;
SNRIs: serotonin noradrenalin reuptake inhibitors; SSRIs: selective serotonin reuptake inhibitors;
TCAs: tricyclic antidepressants
